# Challenges and Possibilities in Preventing Emerging Infectious Diseases in Long-Term Care: A Qualitative Descriptive Study

**DOI:** 10.1155/jonm/2732734

**Published:** 2025-10-14

**Authors:** Po-Jen Kung, Ching-Min Chen, Katherine A. Ornstein, Yi-Yuan Cheng

**Affiliations:** ^1^Department of Nursing, National Cheng Kung University, Tainan, Taiwan; ^2^School of Nursing, Johns Hopkins University, Baltimore, Maryland, USA; ^3^Center for Equity in Aging, The Johns Hopkins University School of Nursing, Baltimore, Maryland, USA; ^4^Bloomberg School of Public Health, Johns Hopkins University, Baltimore, Maryland, USA; ^5^National Miaoli Senior High School, Miaoli, Taiwan

**Keywords:** COVID-19, infectious disease, long-term care, pandemic, prevention, qualitative study, thematic analysis

## Abstract

**Background:**

Emerging infectious diseases pose significant risks to vulnerable populations, particularly older adults with underlying health conditions living in long-term care institutions. Yet, while the experiences of healthcare workers in managing these crises are invaluable, there remains limited research on the specific challenges they faced.

**Aim:**

To explore the experiences of long-term care workers—including nurses, nursing assistants, and managers—in preventing emerging infectious diseases, the challenges they faced during the pandemic, and how they addressed them.

**Methods:**

Twelve participants from long-term care institutions in Taiwan (including assisted living facilities, residential care homes, and nursing homes) were recruited via purposive sampling. All participants worked in the sector during the COVID-19 pandemic, were actively involved in clinical care or institutional management, and were proficient in Mandarin. In-depth interviews were conducted face-to-face between July and August 2024, and data interpretation was guided by thematic analysis.

**Findings:**

Four themes emerged. Two themes depicting challenges illustrate challenges arising from inadequate preparation, including technical, cultural, and communication barriers, noncompliance among family members, resource shortages, and the physical and emotional strain endured by frontline workers balancing duty with feelings of guilt and anxiety. Two other themes representing the possibilities for strengthening long-term care highlight the importance of ongoing education, simulation-based training, and fostering participation, as well as the need for adaptive surveillance and tailored crisis response plans.

**Conclusion:**

Long-term care workers reported systemic vulnerabilities, including inadequate preparation, communication barriers, and emotional strain. They also emphasized the importance of inclusive training, simulation-based practice, and adaptive response strategies.

**Implications for Nursing Management:**

Long-term care institutions should strengthen preparedness by incorporating multilingual, simulation-based infection prevention training, developing family communication strategies that balance clear rationales with dialog, establishing routine surveillance with adaptive response plans, and ensuring psychosocial support for their workforce.

## 1. Introduction

Emerging infectious diseases have become a major threat to global health security. According to the National Institute for Occupational Safety and Health [1], emerging infectious diseases are infectious diseases whose incidence has increased in the past two decades or threatens to increase in the near future, and which do not adhere to national boundaries. Coronavirus disease 2019 (COVID-19) is one such disease—its potential for rapid transmission is what makes it particularly dangerous, as evidenced during the pandemic. When the pandemic broke out in 2019, 693,224 patients worldwide were found to be infected with the virus within about 100 days after the initial outbreak [2], indicating that the virus could spread rapidly via person-to-person contact, with potentially high reproductive numbers [3]. By the end of June 2023, more than 750 million people worldwide had been infected with COVID-19, resulting in more than 6.9 million deaths [4]. The rapid spread of this virulent infectious disease surpassed what many scientists had thought possible, and the emergence of rapidly evolving mutant viral strains such as Alpha, Delta, Omicron, and the BA-series presented health authorities and governments with major challenges in developing effective strategies to combat the pandemic.

Long-term care institutions were among the hardest-hit settings. During the early phase of the COVID-19 pandemic, specifically March to May 2020, residents of long-term care institutions across many European countries accounted for approximately 30% to 60% of all COVID-19 deaths [5]. The disproportionate mortality reflects the risk profile of long-term care residents: advanced age and multimorbidity are common and are associated with severe disease and death from COVID-19 [6–8], and most residents of long-term care institutions are of advanced age and have underlying diseases that render them particularly vulnerable [9]. In addition, the close contact and hands-on nature of care in congregate settings can facilitate transmission, further elevating risk for this population.

Beyond resident vulnerability, institutional and workforce factors magnified risk. Long-term care institutions faced a wide range of challenges, including staff shortages, limited infection prevention and control resources, constantly shifting guidelines, limited opportunities for training, and poor communication. Gaps in disease-prevention knowledge and preparedness were also widely reported among frontline workers. For example, uncertainties surrounding disease progression among long-term care residents left some workers feeling that the care they provided fell short of professional competencies [10], while the level of knowledge regarding emerging infectious disease prevention among long-term care nurses was found to be inadequate [11]. These systemic and workforce challenges underscored structural vulnerabilities within long-term care institutions and have prompted calls for sector-wide reforms [12, 13].

Yet, despite increased attention to infection control in long-term care during the COVID-19 pandemic, few studies have examined the issue through the lens of frontline workers' experiences. This qualitative descriptive study addresses that gap by exploring the firsthand experiences of workers in long-term care institutions with regard to the prevention of emerging infectious diseases, with the aims of identifying and characterizing key challenges, documenting strategies used during the COVID-19 pandemic, and yielding actionable suggestions—including training needs—to strengthen preparedness and better support the workforce of these institutions.

## 2. Materials and Methods

### 2.1. Research Design

This exploratory study adopted a qualitative descriptive design, which is appropriate for investigating understudied real-world issues, as it provides direct, minimally theorized accounts of participants' experiences [14, 15].

### 2.2. Study Participants

Long-term care institutions (including assisted living facilities, residential care homes, and nursing homes) were selected in Taiwan via convenience sampling. Participants (*n* = 12; 4 nurses, 4 nursing assistants, and 4 managers) were recruited via purposive sampling for in-depth interviews. Requirements included experience working in long-term care institutions during the COVID-19 pandemic, current employment in clinical care or institutional management, and proficiency in Mandarin. These criteria were intended to ensure that participants had direct, relevant experience with institutional pandemic prevention. Including multiple staff roles allowed the study to capture insights from both frontline and administrative perspectives. Mandarin proficiency was essential for conducting interviews without language barriers. One participant was recruited from each of 12 long-term care institutions (4 nursing homes, 4 residential care homes, and 4 assisted living facilities) located across Northern, Central, Southern, and Eastern Taiwan, encompassing both urban and rural areas, to support transferability through basic geographic diversity. All participants provided written informed consent. A total of 12 interviews were conducted, based on the recommendation of Guest et al. [16] who noted that a minimum of 12 interviews is generally sufficient to reach data saturation in qualitative research. In this study, data saturation was achieved after the 12 interviews were conducted, as no new themes emerged thereafter.

### 2.3. Data Collection

The time and location of interviews were selected by the participants to ensure that they felt comfortable with the arrangement and to protect their privacy. The interviews, conducted in Mandarin, were audio-recorded with their consent and transcribed within 2 days. On average, each interview lasted approximately 45 to 60 min. The research team conducted debriefing sessions after each interview to discuss emerging themes and to ensure data consistency. In addition, field notes were taken during and after interviews to record nonverbal cues and contextual observations; these notes were later reviewed and integrated into the thematic analysis to enrich interpretation. Semistructured interview guidelines were developed based on the study's objectives and the findings of a recent scoping review on health and care workers in long-term care facilities and their role in preventing emerging infectious diseases [17]. These guidelines were also refined through discussions with experts in long-term care, infection control, and qualitative research. Interview questions included the following: (1) What effective prevention strategies did your affiliated institution employ during the COVID-19 pandemic? (2) What challenges did your affiliated institution face in implementing pandemic prevention measures at the peak of the COVID-19 pandemic? (3) How did your affiliated institution overcome pandemic prevention–related challenges during the pandemic? (4) What suggestions do you have for your affiliated institution regarding the prevention of future emerging infectious diseases? and (5) What training would enhance staff competency in preventing emerging infectious diseases, including knowledge, attitude, and skills?

The interviews were conducted by a doctoral nursing student trained in interview skills and qualitative methods, who had no supervisory or employment relationship with the participants and no affiliation with any of the participating institutions. To maintain reflexivity, we used bracketing and brief pre- and postinterview reflexive memos. In addition, reflexive notes informed minor probe refinements during data collection and codebook updates during analysis. Open-ended questions were used to obtain detailed information on the perspectives of health and care workers in long-term care institutions. The interviews took place between July and August 2024. Preliminary analytic insights were recorded during data collection. However, final themes were developed after full coding. Data collection and analysis proceeded iteratively: after each interview, the team prepared a rapid summary, drafted an analytic memo, and held a short debrief; early insights informed subsequent interviews by refining probes and wording, while the guide's core domains remained unchanged.

### 2.4. Data Analysis

We used constant-comparative techniques to examine similarities and differences within and across cases, updating a shared codebook through iterative team discussions. This study used thematic analysis to analyze the interview transcripts and field notes by themes and subthemes [18]. Data analysis was conducted in the following steps: (a) achieving familiarity with the data through open-minded reading; (b) searching for meanings and themes; and (c) organizing themes into a meaningful whole. The NVivo software program (Version 14.23.0) was used. The two authors coded and analyzed the interview transcripts independently, and the final coding themes and quotations were sent to the other qualitative research expert on the team for content validation. This method of multiple coding was effective in ensuring rigor in qualitative research [19]. A total of 70 initial codes were generated across the 12 interview transcripts. These codes were iteratively compared, discussed, and grouped into nine subthemes, which were then synthesized into four overarching themes—two reflecting institutional challenges and two highlighting future possibilities. The subtheme and theme development process involved multiple rounds of discussion between the two primary coders and the qualitative research expert to ensure clarity, coherence, and alignment with participants' narratives. Finally, the results were presented to the participants to read and review to ensure that the results of the data analysis accurately reflected their actual experiences.

### 2.5. Ethical Considerations

Ethical approval for this study was obtained from the Human Research Ethics Committee of National Cheng Kung University (Approval No. NCKU HREC-E-113-248-2) on May 22, 2024, and the first participant was recruited in June. The objectives, risks, and potential benefits of participation in the study were explained to potential participants, as well as the fact that participation in the study was completely voluntary. Adequate time was provided to potential participants to make the decision of whether to participate in the study before they were asked to sign an informed consent form. Participants were free to withdraw at any point in the study without giving a reason. All interview data were treated anonymously and used strictly for research purposes only.

### 2.6. Study Rigor

In order to ensure rigor in data collection and analysis, this study followed the four core quality criteria of qualitative research: credibility, transferability, dependability, and confirmability [20]. To ensure credibility, the interviews were audio-recorded and subsequently converted into verbatim transcripts by the first author, after which each transcript was returned to the corresponding participant for verification and clarification—a process of member checking to ensure the accuracy and intended meaning of the data. For dependability, two authors independently coded and analyzed the transcripts, and a third team member—an experienced qualitative researcher who was not involved in the initial coding—reviewed the final themes and quotations as an inquiry auditor to enhance analytic reliability. Confirmability was supported by maintaining an audit trail of raw data, transcripts, coding records, and analytic memos, allowing for transparency and external review. Lastly, to ensure transferability, participants were recruited among nurses, nursing assistants, and institution managers with practical experience in clinical care or institution management via purposive sampling. Triangulation was achieved through diverse participant roles (data triangulation) and the involvement of multiple researchers in the analysis (investigator triangulation), thereby enhancing the richness and trustworthiness of the findings. In addition, this study adhered to the Consolidated Criteria for Reporting Qualitative Research (COREQ) checklist to ensure transparency and completeness in reporting [21].

## 3. Results

The demographics of the participants are detailed in Table 1. A total of 12 participants were interviewed, including four nurses, four nursing assistants, and four managers, with one male nursing assistant and the remaining participants being female. The average age of the participants was 37.3 years. In terms of educational background, one nursing assistant held a high school diploma, one nurse and one nursing assistant had associate degrees, and the remaining participants possessed bachelor's degrees. The average total work experience in healthcare services was 13.6 years, with an average of 10.2 years specifically in long-term care institutions. Notably, even the participant with the least experience had worked for a minimum of 3 years in the field. The analysis generated four themes related to emerging infectious disease prevention in long-term care institutions: two themes depicting challenges—“Islands Amid the Storm” and “Cracks in the Armor: Frontline Fragility”—and two forward-looking themes representing possibilities for improving prevention efforts in long-term care—“Enhancing Stability Through Preparedness and Participation” and “Building a Shield in Uncertain Times.” These themes and their subthemes, along with brief descriptions, are presented in Table 2 to enhance transparency and illustrate the analytical depth and structure derived from the coding process.

### 3.1. Theme 1: Islands Amid the Storm

Long-term care institutions, often isolated from the robust infrastructure of acute care systems, found themselves particularly vulnerable during the pandemic. This theme captures the compounding pressures caused by systemic neglect, communication barriers, and external misunderstandings.

One key challenge was insufficient preparation—both technical and cultural. Taiwan's heavy reliance on foreign nursing assistants became a point of fragility, as many struggled to apply infection control principles due to linguistic barriers and unfamiliar care expectations. As one nursing assistant explained, “It wasn't just about learning the necessary skills anymore—it was about applying them under intense pressure while also bridging the communication gap” (#08). Limited training and lack of shared cultural context made it difficult for staff to recognize risks and respond effectively.

At the same time, institutions faced intense pressure from family members, many of whom resisted strict infection prevention measures. While intended to protect residents, rules such as visitation limits or virtual alternatives were often misinterpreted as callous and uncaring. A manager described how “countless hours” were spent responding to family complaints and enforcing rules, while a nurse recounted how some visitors “blatantly broke the rules—taking off residents' masks, secretly feeding them food” (#01).

These challenges were further compounded by critical shortages of protective supplies, including personal protective equipment (PPE). Unlike hospitals, long-term care facilities were deprioritized in supply distribution. One manager expressed the frustration clearly: “We even reported our difficulties with PPE to health departments, but nothing changed” (#12). Staff frequently resorted to purchasing their own masks, straining morale and highlighting systemic inequities.

### 3.2. Theme 2: Cracks in the Armor: Frontline Fragility

The pandemic exposed the physical and emotional vulnerabilities of frontline staff in long-term care institutions. Health and care workers endured strenuous working conditions, intensified by the constant demands of infection control and the psychological toll of high-risk caregiving. Prolonged use of PPE led to exhaustion, discomfort, and visible physical strain, while frequent testing procedures caused additional bodily pain. As one nurse recalled, “After an 8 to 12-hour shift, I would be soaked in sweat, and my face would have deep marks of the N95 mask” (#04). Another described the pain from daily nasal swabs, saying, “I was always very close to bursting into tears” (#07).

Beyond physical discomfort, many workers carried an emotional burden rooted in fear, guilt, and moral distress. The persistent anxiety of becoming infected—or worse, transmitting the virus to their own families—was ever-present. At the same time, when colleagues tested positive, those remaining had to absorb the increased workload, leading to burnout. “Taking care of COVID-positive residents was terrifying,” said one nurse. As she spoke, her voice trembled and she avoided eye contact—revealing the lingering psychological toll of the ordeal (#10). Another nursing assistant added, “Whenever my colleagues tested positive, the workload doubled. After work, I was too exhausted to do anything but lie down” (#11).

The relentless pace of care also left workers questioning their own professional adequacy. Some struggled with guilt over being unable to respond promptly to residents' needs due to overwhelming demands. One nursing assistant explained, “I'd report the residents' symptoms, but the nurses were too overwhelmed to administer medications in time. Seeing residents suffer so much made me feel sorry for them—the guilt was unbearable” (#02). The emotional strain blurred the boundary between professional responsibility and personal anguish, contributing to lasting psychological fatigue.

### 3.3. Theme 3: Enhancing Stability Through Preparedness and Participation

To foster resilience in the face of future health crises, long-term care institutions must go beyond emergency responses and invest in cultivating a stable, engaged, and knowledgeable workforce. A key strategy highlighted by participants was ongoing, inclusive education. Staff advocated for infection control training that was not only consistent and up-to-date, but also tailored to the diverse linguistic backgrounds of the workforce. One nursing assistant proposed, “Since my colleagues and I come from different countries, I've suggested creating infection control materials in multiple languages so that everyone could understand them easily” (#05). Another manager emphasized the importance of offering learning opportunities to all staff levels: “Previously, only nurses were sent to external courses. I'd recommend opening the opportunity to outstanding nursing assistants as well” (#09).

In addition to theoretical instruction, participants stressed the value of hands-on simulation and scenario-based training. Repeated drills—especially those involving role rotation—enabled staff to internalize protocols, practice clear communication, and anticipate responses under pressure. One nurse noted, “Simulation-based training may help everyone understand the pandemic protocols, communication process, and our own roles. It could prevent chaos when actual confirmed cases emerge” (#04). Another manager recommended incorporating drills focused on emerging diseases like Mpox and reviewing each session to support ongoing improvement (#03).

Beyond skills and protocols, participants emphasized that psychological engagement—feeling valued, recognized, and empowered—was essential for maintaining motivation during prolonged crisis periods. Acts of recognition, whether symbolic or practical, had a significant impact. One nursing assistant reflected, “When the pandemic was at its peak, our team leader would bring us herbal tea and snacks… it gave me the energy to keep going” (#11). A manager similarly advocated for institutional mechanisms to boost morale, such as award systems: “I believe it fostered a sense of pride and created a positive atmosphere in the team” (#12).

These accounts reveal that preparedness is not only a matter of resources and protocols, but also of culture. Institutions that foster shared purpose, mutual learning, and emotional reinforcement are better positioned to sustain effective infection control over time.

### 3.4. Theme 4: Building a Shield in Uncertain Times

During unpredictable outbreaks, long-term care institutions must respond rapidly and decisively. This theme captures the operational urgency and strategic flexibility required to protect residents and staff amid evolving threats. Participants described how daily routines were upended as facilities implemented real-time surveillance and strict access controls to reduce infection risks. One manager shared, “We followed the Central Epidemic Command Center's press releases closely, tracked overlaps in confirmed cases, and assessed those with suspected symptoms” (#06). During the interview, she appeared visibly fatigued and sighed repeatedly as she spoke—embodying the ongoing strain of having to constantly adjust to shifting pandemic protocols. Surveillance also extended to visitors, with institutions enforcing registration systems, TOCC screening, and symptom checks. “Strict visitor management was the way to go,” one nurse explained, “limiting numbers, requiring real-name registration, and monitoring temperatures” (#07).

Rapid changes in testing technologies and viral variants also demanded ongoing adaptation. Staff had to master new procedures quickly—such as shifting from PCR to rapid antigen tests—and internalize updated definitions of close contact and exposure. “We had to continually update our prevention measures,” a manager noted, emphasizing that “educating everyone on the latest protocols was key to stopping the spread” (#03). One nurse added, “Later, home rapid antigen test kits became accessible, so we had to learn how to use them quickly to detect confirmed cases earlier” (#01).

Institutional agility was equally vital at the strategic level. Participants described how outbreak severity dictated the phased activation of tailored crisis response plans. These included suspending in-person visits, increasing disinfection frequency, managing residents' dining arrangements, and restricting off-hour gatherings. “We prepared and allocated protective supplies, set usage guidelines, and activated our response plan step by step according to the facility's risk level,” said a manager (#09). Such structured flexibility allowed institutions to respond in real time while maintaining stability in core care operations.

## 4. Discussion

This study explored long-term care workers' experiences in preventing emerging infectious diseases during the COVID-19 pandemic, with a focus on the challenges they encountered and the strategies employed to overcome them. By incorporating both frontline and managerial perspectives, it addresses a persistent gap in the literature and offers a more nuanced account of how prevention was implemented in practice, especially within the understudied long-term care sector. The findings allow decision makers to draw on valuable insights from those most directly involved in prevention efforts in long-term care settings to identify opportunities for enhancing preparedness and devise training and support systems that truly reflect their needs, challenges, and environments.

The unique burdens these workers encountered during the pandemic revealed areas in urgent need of improvement. Due to severe resource and labor shortages, there is a pressing need to develop more effective staffing and workload management strategies, which, in turn, would improve the quality of care delivered [22]. Inadequate staff preparedness, which complicated the already challenging task of infection control, highlights the importance of ongoing training and a supportive environment that promotes learning during routine operations, as well as the timely activation of emergency response plans during crises. Communication barriers—especially those among foreign nursing assistants—highlight shortcomings in current training systems and demonstrate the need for multilingual training tailored to a linguistically and culturally diverse workforce. Moreover, the findings suggest a potential overreliance on foreign nursing assistants in Taiwan, which represents a critical issue warranting further investigation, given its long-term implications for workforce sustainability and care quality.

Another key issue highlighted was the extreme isolation and resource constraints faced by long-term care institutions during the pandemic. Many respondents reported persistent shortages of PPE, a critical issue throughout the crisis [23, 24]. Resource allocation from the government and private sector predominantly favored hospitals, leaving long-term care institution staff particularly vulnerable due to systemic inequalities [25]. This lack of support often forced staff to procure their own protective gear, underscoring the institutions' inability to adequately safeguard their workforce. Such conditions likely contributed to staff turnover, which further added to the strain on an already insufficient workforce [26].

The multicultural composition of the workforce also introduced unique challenges, such as language barriers and differing cultural norms related to hygiene and caregiving practices, which complicated resident care and heightened the risk of conflicts with residents and their families [27, 28]. Tensions between long-term care institutions and residents' family members—particularly regarding infection prevention protocols—hindered infection control and prevention efforts. Participants described instances in which families expressed anger, mistrust, or outright noncompliance in response to visitation restrictions and quarantine measures. This phenomenon echoes findings from another study showing that families often perceived pandemic-related rules as alienating or emotionally harmful to their loved ones [29].

These dynamics are especially salient in the Taiwanese context—where this study was conducted—given the cultural emphasis on filial piety and the expectation of close familial involvement in elder care. Within such a cultural framework, strict institutional policies may be perceived not only as necessary precautions but also as emotionally neglectful or morally unjust, thereby intensifying family resistance. Moreover, such communication challenges during the COVID-19 pandemic further revealed critical gaps in family inclusion within long-term care settings. Inadequate communication and exclusion of family members from decision making led to emotional distress and, in some cases, resistance among family members [30]. Similarly, Cohen et al. [31] found that when families were excluded from key decisions, feelings of anger, sadness, and injustice intensified, with some resorting to direct confrontation with institutional staff. These reactions should not be dismissed as mere noncompliance, but rather understood as expressions of emotional distress and disrupted caregiving expectations. Our research team recommends that future outbreak preparedness plans in the long-term care sector adopt family-centered communication strategies—including transparent explanations of infection control measures, structured opportunities for dialog, and culturally attuned support for family caregivers.

A recent review of the experiences of long-term care institution workers during the COVID-19 pandemic also highlighted increasing job insecurity and social marginalization within these institutions, which further exacerbated workplace inequalities and intersectional racism [32]. These findings are consistent with the review conducted by Lightman [33] who argued that research must address such inequalities in long-term care to better support workers both during and after the pandemic. Prioritizing cultural safety and dismantling systemic barriers that contribute to mental distress among workers is essential for maintaining the quality of resident care.

Furthermore, the mental and emotional toll of the pandemic on staff was evident throughout our interviews, underscoring the urgent need for comprehensive mental health and psychosocial support in long-term care institutions. The World Health Organization [34] highlighted the potential negative impact of the COVID-19 pandemic on the mental well-being of healthcare workers, with reports of trauma and stress from frontline workers [35]. Many healthcare workers reported feeling blamed for “bringing the virus into the long-term care institution from their community” [33]. Similarly, participants in this study expressed anxiety over their dual responsibilities: caring for infected residents and protecting their own families. The fear of contracting the virus and transmitting it to loved ones was a psychological barrier many struggled to overcome. Addressing these concerns is essential to supporting staff and protecting their well-being during future outbreaks, and national infection control standards and policies should be aimed at prioritizing staff protection and ensuring that appropriate measures are taken to provide a safe and healthy work environment.

However, despite these challenges, our interviews also revealed a sense of foresight among long-term care institution staff regarding the future of emerging infectious disease prevention. They shared forward-looking ideas and concrete strategies, which could be broadly categorized into “routine measures (Enhancing Stability Through Preparedness and Participation)” and “emergency response strategies (Building a Shield in Uncertain Times).” Our findings suggest that long-term care institutions should routinely carry out diversified infection control training, develop multilingual training materials tailored to staff from different countries, and implement “dynamic training that evolves based on actual circumstances” during actual outbreaks. The importance of training was also highlighted in a study of 720 Portuguese long-term care staff, where 70% identified staff training as crucial for ensuring well-being and fostering a safety culture [36]. Strengthening “scenario-based training (Scenario-Based Simulation and Practice)” also emerged as a key theme, which was reported as useful in reducing uncertainty and panic during real-world outbreaks. This is consistent with the results of a survey conducted by Kung et al. [17] which identified emergency drills as a primary predictor of prevention competency. Moreover, while staff were aware of the necessary preventive measures, many reported feeling inadequately prepared due to a lack of hands-on experience with specific protocols. This indicates the importance of practical training to build confidence and competence.

Our study also identified a gap in training opportunities for nursing assistants. Despite their essential role in long-term care institutions, nursing assistants are often overlooked when it comes to professional development opportunities. Addressing this gap is crucial for recognizing their contributions and equipping them with the skills needed to effectively support infection prevention efforts. Feeling recognized and valued has been found to be critical for motivating staff and fostering a positive work environment, and such positive reinforcement can protect workers from burnout and enhance overall resilience [37–39].

Addressing the aforementioned challenges will require strong leadership and significant improvements in working conditions [25]. We believe that it is important for long-term care institutions to implement holistic support systems that address the physical, mental, and emotional needs of their staff, and that providing such support is not merely a protective measure but an ethical imperative of the healthcare industry, as well as a practical necessity to ensure the sustainability of long-term care services during public health crises.

## 5. Limitations

In this study, participants had an average of ten or more years of work experience, and their perspectives may not be generalizable to those of less experienced workers. Future studies may wish to consider how varying levels of experience impact opinions and perceptions regarding emerging infectious disease prevention.

Additionally, because proficiency in Mandarin was a requirement for participation, all participants were Taiwanese nationals working in long-term care institutions. While their insights provide valuable and culturally specific perspectives, it is worth noting that foreign nursing assistants, who make up a significant portion of the workforce, may face unique challenges. As indicated by the findings of this study, pandemic prevention in long-term care institutions also involves addressing cultural and language barriers encountered by workers of different nationalities.

Furthermore, as the interviews relied on participants' recollections, there is a potential for recall bias.

Finally, while the qualitative nature of this study provides rich, contextual insights, it lacks the statistical generalizability offered by quantitative approaches. Previous research has quantitatively assessed emerging infectious disease prevention in long-term care settings, including the development and validation of competency assessment instruments and national surveys that identified key predictive factors. These studies have established a foundational understanding of the current state of pandemic prevention competencies. Building upon these insights, this qualitative study fills a critical knowledge gap by providing insights into effective policies and procedures implemented during the COVID-19 pandemic.

## 6. Conclusions

This study highlights the pivotal role of long-term care institutions during outbreaks of emerging infectious diseases, as well as the challenges they faced during the recent COVID-19 pandemic—emphasizing both the sector's invaluable contributions and its profound vulnerabilities. Our findings indicate an urgent need to address longstanding systemic challenges so as to mitigate the impact of future pandemics. We believe that the outcomes of this study will contribute significantly to managing future pandemics in long-term care settings. The participants' experiences reveal significant barriers faced by the institutions they worked in and offer concrete, actionable recommendations grounded in the firsthand experiences of frontline workers, which serve not only to inform routine practices but also enhance emergency response strategies. It is our hope that policymakers and long-term care administrators can harness these insights to better support staff and residents during future outbreaks and refine pandemic prevention protocols to enhance preparedness and resilience in long-term care institutions.

## Figures and Tables

**Table 1:** Demographic characteristics of the interviewees (*n* = 12).

No	Job role	Gender	Educational level	Age	Years of service in long-term care institutions	Cumulative years of experience in healthcare services
#01	Nurse	Female	Junior college	43	20	21
#02	Nursing assistant	Female	University	33	10	10
#03	Manager	Female	University	40	10	15
#04	Nurse	Female	University	24	3	3
#05	Nursing assistant	Female	High school	28	3.5	3.5
#06	Manager	Female	University	38	7	15
#07	Nurse	Female	University	38	15	18
#08	Nursing assistant	Male	Junior college	30	6	8
#09	Manager	Female	University	53	13	28
#10	Nurse	Female	University	37	10	15
#11	Nursing assistant	Female	University	40	5	6
#12	Manager	Female	University	43	20	21

**Table 2:** Challenges and possibilities in emerging infectious disease prevention in long-term care institutions.

Theme	Subtheme	Brief description
Islands amid the storm	Fragile infrastructure and workforce	Long-term care institutions faced systemic shortages in staffing and equipment—an issue compounded by weak integration with acute care systems.
	Cultural and communication gaps	Foreign workers faced difficulty understanding infection control procedures due to language barriers and differing cultural norms regarding hygiene and caregiving practices.
	Difficulty with family cooperation	Families often resisted infection prevention measures, resulting in emotional labor for workers and rule violations.

Cracks in the armor: Frontline fragility	Physical strain and overwork	Workers endured extreme physical discomfort from prolonged PPE use and increased workloads due to staff shortages.
	Emotional burden and moral distress	Frontline staff experienced fear of infection, helplessness, and guilt due to being unable to provide inadequate care during crises.

Enhancing stability through preparedness and participation	Inclusive and ongoing education	Ongoing infection control training—supported by multilingual materials—ensured that all staff were equipped with essential knowledge.
	Scenario-based simulation and practice	Regular pandemic drills and role rotations improved team coordination and preparedness under pressure.
	Recognition and team empowerment	Acts of recognition and supportive leadership boosted morale and encouraged active participation in infection prevention efforts.

Building a shield in uncertain times	Real-time monitoring and control	Institutions implemented strict visitor screening, symptom surveillance, and close communication with public health agencies.
	Adaptive training and crisis response	Staff adapted to rapidly changing protocols, adopted new testing tools, and activated tiered response plans based on outbreak severity.

## Data Availability

The data that support the findings of this study are available from the corresponding author upon reasonable request.
